# Complement Split Products in Amniotic Fluid in Pregnancies Subsequently Developing Early-Onset Preeclampsia

**DOI:** 10.1155/2015/263109

**Published:** 2015-10-18

**Authors:** Manu Banadakoppa, Alex C. Vidaeff, Uma Yallampalli, Susan M. Ramin, Michael A. Belfort, Chandra Yallampalli

**Affiliations:** Department of Obstetrics and Gynecology, Baylor College of Medicine, Houston, TX 77030, USA

## Abstract

*Objective*. To determine the second-trimester amniotic fluid concentrations of complement split products in pregnancies subsequently affected by early-onset preeclampsia. *Study Design*. Cohort of 731 women with singleton pregnancies undergoing second-trimester genetic amniocentesis followed up to delivery and analyzed as a nested case-control study. Cases of preeclampsia developing before 34 weeks' gestation (*n* = 15) were compared with 47 uncomplicated term controls. Amniotic fluid collected at amniocentesis was tested for complement split products Bb, C4a, C3a, and C5a. *Results*. Women who developed early-onset preeclampsia as compared with the term pregnant controls had significantly higher (*P* = 0.04) median amniotic fluid C3a levels (318.7 ng/mL versus 254.5 ng/mL). Median amniotic fluid Bb levels were also significantly higher (*P* = 0.03) in preeclamptic women than in normal pregnant women (1127 ng/mL versus 749 ng/mL). Median levels of C4a and C5a were not significantly different between the groups. *Conclusion*. Our data suggest that complement activation in early pregnancy is associated with early-onset preeclampsia. We believe this to be the first prospective study to link complement activation in amniotic fluid in early pregnancy and later development of preeclampsia. Our findings provide evidence that immune dysregulation may precede the clinical manifestations of preeclampsia and that the alternative complement pathway is principally involved.

## 1. Introduction

Preeclampsia is a pregnancy-specific heterogeneous condition. Although typically diagnosed after 20 weeks' gestation, it is considered that preeclampsia begins much earlier in pregnancy in connection with abnormal placental development [1]. Its clinical manifestations range from mild to severe and it has been suggested that preeclampsia may have 2 different phenotypes, early- and late-onset preeclampsia, depending on whether the clinical manifestations began before or after 34 weeks' gestation [2]. The early form is characteristically associated with fetal-placental unit impairment (fetal growth restriction, placental functional abnormalities), while the late-onset form presents a mixture of maternal clinical manifestations but only moderate-to-absent placental dysfunction. This difference in presentation suggests different pathogenic pathways [3].

Studies performed nearly 30 years ago found marked elevations in levels of complement factor B, C3, C4, and C5 split products in chorionic villi from preeclamptic pregnancies indicating excessive classical and alternative pathway activation [4]. Since then, there has been accumulating evidence for a role of the complement system activation in the pathogenesis of preeclampsia [5–7]. Recent investigations have detected more pronounced associations between complement activation and early-onset preeclampsia than late-onset preeclampsia and suggested a more homogeneous pathogenesis in early-onset preeclampsia [8].

Although most studies have focused on systemic maternal complement activation, complement activation products have also been demonstrated in fetal circulation, decidua, chorionic villi, and placental vascular walls in women with preeclampsia, supporting local complement activation with placenta targeting as a mechanism of interest for preeclamptic placental pathology [9]. Investigating complement activation locally, in the placenta and amniotic fluid, is an excellent strategy to determine events occurring at the fetal-maternal interface. Consequently, the present study aimed to determine the second-trimester amniotic fluid concentrations of complement split products Bb, C4a, C3a, and C5a in pregnancies subsequently affected by early-onset preeclampsia compared to control uncomplicated pregnancies. We hypothesized that complement activation would be detectable early in the pathogenic pathway to preeclampsia.

## 2. Materials and Methods

The study cohort included a select group of women with singleton nonanomalous pregnancies undergoing second-trimester genetic amniocentesis between 15 and 26 weeks' gestation. The subjects were prospectively enrolled from June 2007 through November 2009 at multiple centers in Greater Houston area, Texas, followed up to delivery, and analyzed as a nested case-control study. Subjects were excluded from the analysis if the amniocentesis results indicated abnormal fetal karyotype or intra-amniotic infection, or if the detailed ultrasound preceding amniocentesis revealed major fetal anomalies, oligohydramnios, or early fetal growth restriction.

In accordance with the definitions set by the Hypertension in Pregnancy document developed by the American College of Obstetricians and Gynecologists Task Force on Hypertension in Pregnancy in 2013 [10], 15 study participants were ultimately diagnosed with early-onset preeclampsia (before 34 weeks). The diagnosis was established by the treating physician and confirmed by the principal investigator (ACV) after review of the hospital computerized record. All cases delivered before 36 weeks' gestational age. Control women (*n* = 47) were selected on the basis of having an uncomplicated, normotensive pregnancy with a normal term delivery. The control group was matched for gestational age at amniotic fluid collection in order to reduce the potential confounding effect of gestational age on complement factors concentration. Demographic characteristics and pertinent maternal medical and obstetrical history were recorded at the time of enrollment by the genetic counselor and the physician performing the amniocentesis based on a structured data collection form. The circumstances of delivery were determined by review of the hospital computerized record verified by telephone interviews with the enrolled women and/or their primary obstetricians if necessary. Gestational age was determined by known last menstrual period if consistent with fetal biometry at the time of amniocentesis, or by first-trimester sonogram if last menstrual period was unsure or there was more than 7-day difference between menstrual and ultrasound dates. It is known that menstrual dating may systematically overestimate gestational age by one week. For this reason, we selected as controls only women who delivered at 38 weeks' gestation or later to ensure that control deliveries were indeed at term.

At the time of genetic amniocentesis, the first 2 mL of amniotic fluid, routinely discarded, was collected for study purpose and transported in a capped sterile tube to the laboratory where the sample was centrifuged for 10 minutes at 4°C. Visually bloody samples were excluded from processing. The supernatant was aliquoted and stored at −80°C until assay. Levels of complement split products in amniotic fluid were determined using specific and sensitive enzyme-linked immunoassays by a technician blinded to the group assignment. C3a, C4a, and C5a levels were measured using C3a-desArg, C4a-desArg, and C5a des-Arg BD optiEIA ELISA kits, respectively (BD Biosciences, San Jose, CA), according to the manufacturer's instructions. Factor Bb levels were measured using MicroVue Bb plus ELISA kit (Quidel Corporation, San Diego, CA) according to the manufacturer's instructions.

The level for complement split factors Bb, C4a, C3a, and C5a was the main explanatory variable of interest. Differences in medians between the preeclampsia group and the uncomplicated pregnancy control group were tested using the Mann-Whitney test. The difference in the median values between two groups was considered significant if *P* < 0.05. Demographic differences between continuous variables were examined using the *t*-test and differences between categorical variables were assessed using *χ* ^2^, Fisher exact, and Pearson *χ* ^2^ test as appropriate. The statistical software package used was STATA 10.0 (STATA Corp, College Station, TX).

The collection of amniotic fluid samples and the prospective follow-up of enrolled pregnancies was approved by the Institutional Committee for the Protection of Human Subjects at the University of Texas Houston Medical School (HSC-MS-07-0109).

## 3. Results

The original cohort comprised 731 pregnant women undergoing genetic amniocentesis between 15 and 26 weeks' gestation. Excluded from the analytic dataset after enrollment and amniocentesis were 22 cases (8 cases of trisomy 21, 2 of trisomy 18, 1 of monosomy X, 1 case of Klinefelter syndrome, 1 case of deletion 9p24, 1 case of cytomegalovirus intra-amniotic infection, 6 cases lost to follow-up, 1 case of incorrect data recording, and 1 inadequate sample).

The prevalence of early-onset preeclampsia was 2.4% (*n* = 17). Of all these cases, 15 had amniocentesis between 16 and 18 weeks' gestation, one at 21, and still another one at 24 weeks' gestation. The latter two cases were excluded from analysis leaving an index study group of 15 cases. The matched control group (*n* = 47) had amniocentesis performed between 16 and 18 weeks' gestation.

Among cases, delivery occurred between 23 and 36 weeks' gestation. The 3 cases who delivered between 35 and 36 weeks' gestation were diagnosed with preeclampsia without severe features before 34 weeks.

**Figure 1:**
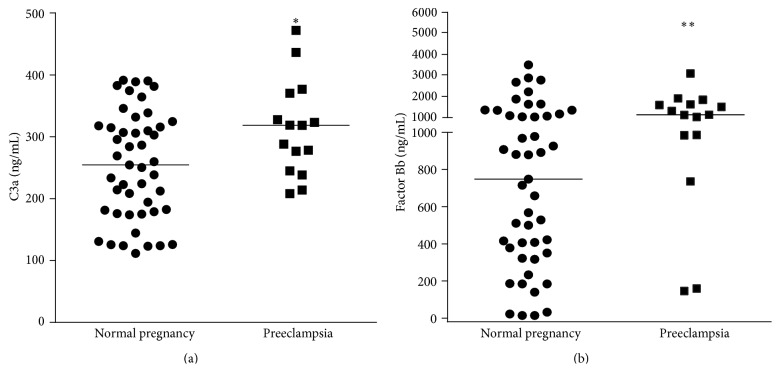
Second-trimester amniotic fluid concentrations of complement split products C3a and Bb. (a) Median level of amniotic fluid C3a was significantly higher (^*∗*^ *P* = 0.04) in women who developed early-onset preeclampsia as compared with the normal term pregnant control group (318.7 ng/mL versus 254.5 ng/mL). (b) Median level of amniotic fluid factor Bb was also significantly higher (^*∗∗*^ *P* = 0.03) in preeclamptic women than in normal pregnant women (1127 ng/mL versus 749 ng/mL).

**Figure 2:**
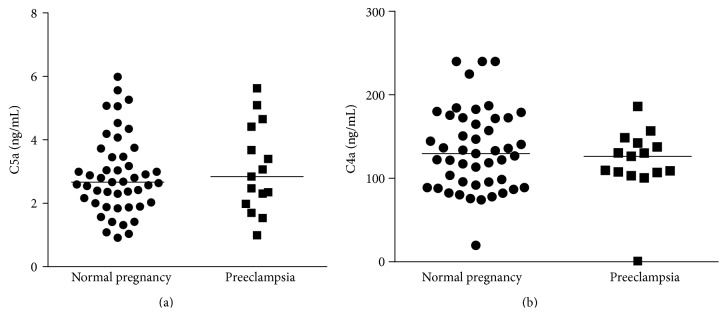
Second-trimester amniotic fluid concentrations of complement split products C4a and C5a. (a) Median levels of amniotic fluid C4a in normal pregnant women (129.6 ng/mL) versus pregnant women who subsequently developed early-onset preeclampsia (126.4 ng/mL) were not significantly different. (b) Median levels of amniotic fluid C5a in normal pregnant (2.663 ng/mL) versus early-onset preeclampsia patients (2.842 ng/mL) were not significantly different.

Complement split products Bb, C4a, C3a, and C5a were identified in all 62 samples tested. The concentration values were not normally distributed. Women who developed early-onset preeclampsia had a significantly higher median amniotic fluid C3a level (318.7 ng/mL; IQR, 244.8–370.4) than those in the uncomplicated pregnancy control group (254.5 ng/mL; IQR, 179.0–317.8; *P* = 0.04). Median amniotic fluid Bb levels were also significantly higher in preeclamptic women (1127 ng/mL; IQR, 984–1624) than in normal pregnant women (749 ng/mL; IQR, 351–1167; *P* = 0.03) (Figure 1). The median levels of C4a and C5a were not significantly different between the groups (126.4 ng/mL versus 129.6 ng/mL and 2.8 ng/mL versus 2.6 ng/mL, resp.) (Figure 2).

Demographic characteristics of patients with early-onset preeclampsia and uncomplicated pregnancies are presented in the table. The subjects in the two study groups were comparable for these characteristics (Table 1).

## 4. Discussion

Our data suggest that increased levels of complement activation as expressed by elevated C3a fragment measured at a single point in amniotic fluid in early pregnancy are associated with subsequent development of early-onset preeclampsia. To our knowledge, this is the first prospective study to examine the relationship between the evidence of complement activation in amniotic fluid in early pregnancy and subsequent development of preeclampsia.

The complement system is a humoral immune amplification system composed of endogenous plasma proteins. Under normal physiologic conditions, activation of complement results in immune cell activation and the rapid opsonization and destruction of pathogens or other “danger signals” [11] such as dying cells, heat shock proteins or in pregnancy, and even apoptotic trophoblast cells [12]. Because complement components are acute phase reaction proteins and pregnancy is a heightened inflammatory state, normal human pregnancy is characterized by systemic complement activation resulting in a significantly increased generation of the split products C4a, C3a, and C5a in the maternal circulation [13]. These glycopeptides, also referred to as anaphylatoxins, are potent immunoinflammatory modulators that bind to their respective receptors to trigger an inflammatory response. The system normally operates at a low steady state; however, excessive activation or inappropriate regulation can lead to even higher maternal plasma concentrations of anaphylatoxins. This unregulated elevation can be injurious and contribute to the pathogenic pathway of pregnancy complications such as early pregnancy loss [14], fetal death [15], preterm birth [16], and preeclampsia [17].

Generally speaking, the biologic functions of the complement system are mediated through the production of activation (or split) fragments, including C3a, C4a, and C5a. Central to complement activation is the formation of a C3 convertase that cleaves C3 into C3a and C3b. C3a is a fluid phase inflammatory product and C3b is a major opsonin and essential part of the C5 convertase that in turn will cleave C5 into C5a and C5b. C3 is an earlier part of the complement cascade than C5, but downstream from C4. As the C4 split products, C4a is still another anaphylatoxin while C4b is a part of the C3 convertase. Bound C4b can be further degraded to C4d, considered to be the most important marker of classical complement activation.

Complement factor B is part of the alternative pathway initiation complex. It is cleaved by factor D into two unequal fragments: Ba and Bb. Bb carries the active site of factor B and will contribute to the cleavage of additional C3 molecules. If increased concentrations of C4 or C4d can be regarded as a marker of complement activation by either the classical or lectin pathways, in contrast, the increased production of Bb characterizes the activation by the alternative pathway.

The association between the complement system and preeclampsia has been addressed in the past with conflicting results. Higher maternal plasma C3a and C5a concentrations have been reported late in pregnancy in preeclampsia and HELLP syndrome [12, 18, 19]. Other reports have found only C5a but not C3a to be elevated in the maternal or fetal circulation [5, 6, 20]. Although earlier reports had suggested that elevations of the anaphylatoxins could not be detected in maternal circulation in the preclinical stage of preeclampsia [5], more recent studies have documented elevations both early and near term [8, 21, 22]. Several factors may explain, at least partly, the conflicting reports in the literature: different methodology, different clinical presentations of preeclampsia, gestational age at assessment, and local versus systemic assessment.

In several studies, the presence of C4d was interpreted as evidence of classical pathway activation at the fetal-maternal interface [23] and placental C4d deposits have been associated with the severity of preeclampsia and significantly lower gestational age at delivery [24]. Besides the classical pathways, the alternative pathway may also be involved in the early complement activation in preeclampsia, as suggested by the elevated complement activation fragment Bb levels in maternal circulation before 20 weeks' gestation [7, 25]. Another example of the confounding effect of the gestational age at testing is that provided by a recent study in which the Bb levels were not increased significantly in plasma of preeclamptic women at term compared with healthy pregnant women, whereas C3a and C4d fragments were significantly increased [12]. Bb is primarily associated with alternative complement activation, whereas C3a can arise from any of the complement pathways.

Haeger et al. were the first to describe the presence of C3a and C5a in amniotic fluid in both preeclampsia and uncomplicated pregnancy shortly before delivery, without differences in concentrations between the 2 groups [18]. It was later confirmed that complement split products C3a, C4a, and C5a are detectable in the amniotic fluid as early as the second trimester, with only C5a showing higher concentrations at term [26]. Others have reported the presence of activation product Bb in the amniotic fluid at concentrations increasing with gestational age [27].

Our findings confirm previous reports of exaggerated complement activation in preeclampsia and provide evidence that immune dysregulation precedes the clinical manifestations of the syndrome. It has been hypothesized that complement activation occurs as a result of ischemia or oxidatively stressed changes in the placenta, consistent features of preeclampsia [12, 28], subsequently triggering a feed-forward cycle of placental damage, antiangiogenic factor production, and maternal vascular damage [29]. The anaphylatoxins are also vasoactive substances that can contribute to pregnancy-induced hypertension [28]. For this hypothesis to be plausible, complement activation has to be documented in the preclinical stage of the disease. Our findings of increased concentration of C3a in amniotic fluid obtained early in pregnancy are consistent with observations made by others early in pregnancy in the maternal circulation [22] and suggest that complement activation plays a significant role even in the subclinical stage of the syndrome and therefore may contribute to preeclampsia in a causal way rather than being a consequence of the systemic inflammatory reaction that characterizes the final clinical stage in the development of preeclampsia.

Studies in mouse models of pregnancy have indicated that complement activation targeted to the placenta drives angiogenic imbalance, placental insufficiency, and endothelial injury [30]. The human fetus can independently synthesize proteins of the complement system [31] and it is possible that the fetus is the main source of the complement system in the amniotic fluid [26]. Our investigation focused on the amniotic fluid, as a possibly better reflection of processes operant at the fetal-maternal interface than maternal plasma. There are instances in human pathology suggesting that peripheral blood complement levels can only partly reflect complement involvement in the disease development process in various target tissues. There may also be potential disparities between peripheral and local complement activation in the same patient [32].

All activation steps in the complement cascade converge on the complement factor C3. Thus, C3 not only seems to be a good indicator for overall complement activation, but may also be of pathophysiological relevance at the fetal-maternal interface. Our findings of increased concentrations of C3a but not C5a in pregnancies subsequently developing early-onset preeclampsia suggest that excessive complement activation does not progress beyond C3 activation in the sample population we studied, possibly because of step-specific regulators in the activation sequence. Complement activation in pregnancy is in part regulated by regulatory proteins localized to villous trophoblast membranes, such as CD46, CD55, and CD59, preventing injurious effects [33]. It has been proposed that, in preeclampsia, expression of these regulators is reduced, leading to excessive complement activation with generation of excess anaphylatoxins and a resulting proinflammatory maternal-fetal environment [20]. Indeed mutations in genes encoding complement inhibitor CD46 are associated with development of preeclampsia, supporting dysregulation of complement activation at least at the CD46 step as a risk factor for preeclampsia [34]. CD46 is a membrane cofactor protein that facilitates degradation of C3b and C4b so that they do not continue as part of the C3 convertase; it is therefore relevant at the C3 activation step. In contrast, CD55 and CD59 are more relevant at the C5 activation step and beyond. In a study that measured placental mRNA expression of the complement regulatory proteins in preeclampsia at the time of delivery, a significant upregulation of CD55 and CD59 mRNA expression was observed [24]. The finding was interpreted as suggesting the existence of local, placental regulatory mechanisms that control downstream activation of the complement system beyond C3a. This is in keeping with our results of higher C3a but not C5a concentrations in amniotic fluid in pregnancies developing preeclampsia.

Limitations of this study include the fact that samples were obtained from a selected population of pregnant women undergoing genetic amniocentesis. Also, inherent to a nested case-control approach is the potential for biases and the inability to draw definitive conclusions regarding causal relationships. Furthermore, the relatively small sample size prevented a stratification analysis by other clinical characteristics. Our database did not capture preexisting medical maternal conditions and no control was provided in analysis for this aspect. However, by study design, we focused on complement split products and their association with the development of early-onset preeclampsia irrespective of the reason behind this or preexisting favoring conditions.

Despite these limitations, the data presented provide additional support for the significant relationship between excessive complement activation in early pregnancy and subsequent development of preeclampsia. It remains unclear what triggers complement activation and which complement pathways are principally involved. Based on our findings (Bb but not C4a elevated) we speculate that there may be stronger links with the alternative complement pathway activation, as previously also proposed by others [21, 35].

Future studies will determine the relative importance of complement activation in the pathogenesis of preeclampsia. In contrast to many other “dead-end” associations studied in relation to the development of preeclampsia, there is some indication that therapeutic manipulations of the complement system during pregnancy-induced hypertensive disorders may be feasible. A recent case report of a woman with preeclampsia/HELLP syndrome receiving eculizumab (the first registered anticomplement drug) described normalization of laboratory values and pregnancy prolongation by 17 days [36]. It is also known that heparin is able to inhibit complement activation [37]. Results of a recent meta-analysis strongly suggest that low-molecular-weight heparin reduces the risk of recurrent placenta-mediated complications including preeclampsia [38]. At experimental level, blocking C3a receptor activation significantly ameliorated key features associated with preeclampsia induced in animal models and impaired placental angiogenesis in cultured human villous explants [39]. Although general complement inhibition may not be an optimal therapeutic strategy in human pregnancy because of the increased risk of maternal and fetal infection, there is hope of identifying new selective immunomodulatory therapeutic agents to advance the treatment of preeclamptic patients.

## Figures and Tables

**Table 1:** Comparison of maternal baseline characteristics between preeclampsia and normal pregnancy groups.

Variable	Preeclampsia (*n* = 15)	Control (*n* = 47)	Significance
Mean maternal age, y	36.1 (SD = 5.9)	36.6 (SD = 4.4)	*P* = 0.74 (Mann-Whitney)
Parity			*P* = 0.79 (*χ* ^2^)
Nulliparous	5 (33.3%)	14 (29.8%)	
Parous	10 (66.7%)	33 (70.2%)	
Race			*P* = 0.49 (Pearson *χ* ^2^)
Asian	2 (13.3%)	16 (34.0%)	
Hispanic	3 (20.0%)	7 (14.9%)	
Black	3 (20.0%)	8 (17.0%)	
White	7 (46.7%)	16 (34.1%)	
Indication for amniocentesis			*P* = 0.19 (Fisher *χ* ^2^)
AMA	9 (60.0%)	35 (74.5%)	
Abnormal screening	4 (26.7%)	4 (8.5%)	
Both AMA and abnormal screening	2 (13.3%)	8 (17.0%)	

AMA, advanced maternal age (≥35 years old).
